# Vitamin D—update for the pediatric rheumatologists

**DOI:** 10.1186/s12969-015-0017-9

**Published:** 2015-05-29

**Authors:** Jelena Vojinovic, Rolando Cimaz

**Affiliations:** Clinic of Pediatrics, Clinical Center, Faculty of Medicine, University of Nis, Bul dr Zorana Djindjica 48, 18000 Nis, Serbia; Dipartimento di Neuroscienze, Area del Farmaco e Salute del Bambino (NEUROFARBA), Viale Pieraccini, 24, 50139 Firenze, Italy

**Keywords:** Vitamin D recommendations, Juvenile idiopathic arthritis, Juvenile systemic lupus erythematosus, D hormone, Pediatric rheumatic diseases

## Abstract

Vitamin D, upon its discovery one century ago, was classified as a vitamin. This classification still greatly affects our perception about its biological role. 1,25(OH)2D (now known as the D hormone) is a pleiotropic steroid hormone that has multiple biologic effects. It is integral to the regulation of calcium homeostasis and bone turnover as well as having anti-proliferative, pro-differentiation, anti-bacterial, immunomodulatory and anti-inflammatory properties within the body in various cells and tissues. Vitamin D (cholecalciferol) should be considered a nutritional substrate that must be ingested or synthesized in sufficient amounts for the further synthesis of the very important regulatory steroid hormone (D hormone), especially in patients with pediatric rheumatic diseases (PRD).

Vitamin D insufficiency or deficiency was shown to be pandemic and associated with numerous chronic inflammatory and malignant diseases and even with increased risk of mortality. Several studies have demonstrated that a high percentage of children with pediatric rheumatic diseases (PRD-e.g., JIA, jSLE) have a vitamin D deficiency or insufficiency which might correlate with disease outcome and flares. Glucocorticoids used to treat disease may have a regulatory effect on vitamin D metabolism which can additionally aggravate bone turnover in PRD. An effort to define the optimal serum 25(OH)D concentrations for healthy children and adults was launched in 2010 but as of now there are no guidelines about supplementation in PRD.

In this review we have tried to summarize the strong evidence now suggesting that as the knowledge of the optimal approach to diagnosis and treatment PRD has evolved, there is also an emerging need for vitamin D supplementation as an adjunct to regular disease treatment. So in accordance with new vitamin D recommendations, we recommend that a child with rheumatic disease, especially if treated with steroids, needs at least 2-3 time higher doses of vitamin D than the dose recommended for age (approximately 2000 UI/day). Vitamin D supplementation has become an appealing and important adjunct treatment option in PRD.

## Background

One century after its discovery and three Nobel prizes awarded for discoveries in this topic [1], we have clear evidences that the so-called vitamin D is in fact a pleiotropic steroid hormone similar to other steroid hormones. Unfortunately, its primary classification into the vitamins still deeply influences our professional perception about 1,25(OH)2D biological function and impact on the occurrence and outcome of the some rheumatic diseases [4]. It is necessary to distinguish cholecaliferol (commonly called vitamin D), a nutritional precursor compound, from the 1,25(OH)2D-vitamin D hormonal form. This hormonal form is synthetized after a complex, endocrine-regulated biochemical process. This 1,25(OH)2D D hormone has its own endocrine, paracrine and autocrine control [5]. As hormone is defined as a chemical substance produced in one part of the body that stimulates functional activity in another part [6], it is clear that what we call vitamin D does not fulfill the vitamin definition criteria but rather those for a hormone. We will discuss the biologic roles of vitamin D in this review as that of the D hormone.

When prescribing medication for the treatment of rheumatic diseases, most of pediatric rheumatologists do not recommend vitamins as mandatory, including vitamin D. Yet newer scientific studies may soon change that approach. Beside regulation of calcium homeostasis and bone turnover, the D hormone has proven antiproliferative, pro-differentiation, anti-bacterial, immunomodulatory and anti-inflammatory properties within the body in various cells and tissues [2, 3]. This effects can be achieved only if the D hormone (1,25(OH)2D) itself, or its agonist, is bound to vitamin D receptor (VDR). The discovery that vitamin D receptor agonists possess immunomodulatory and anti-tumor properties prompted research to investigate the possibility that these agonists might be used as a therapeutic agent for different autoimmune and malignant diseases [7,8].

## Review

Randomized controlled trials providing recommendations for vitamin D supplementation in pediatric patients with PRD are lacking. Yet several recent studies have supported the importance of and necessity for vitamin D supplementation within standard treatment protocols and guidelines of rheumatic diseases in childhood. A study of Arkema et al. [9] investigated the association between UV-B light exposure and the risk of developing rheumatoid arthritis (RA) among women enrolled in two large prospective cohort studies [the Nurses’ Health Study (NHS) and the NHSII]. These studies confirmed a significantly decreased risk of RA with higher UV-B exposure especially between birth and age 15 years. Nisar et al. [10] published a systemic literature review and meta-analysis of current evidence on vitamin D in JIA, summarizing data from 19 papers that were largely supporting positive benefits. Robinson et al. [11] presented data from Atherosclerosis Prevention in Pediatric Lupus Erythematosus (APPLE) trial and found vitamin D deficiency to be common in pediatric lupus and independently associated with elevated hsCRP and an increased cardiovascular disease risk. Finally, Holick reviewed recent recommendations and clinical guidelines [12] and suggested that vitamin D supplementation of up to 2000 IU/d appears to be safe and well tolerated in children with chronic diseases.

### D-hormone synthesis

Cholecalciferol is pre-hormonal form of D hormone that must be ingested or generated in the skin where one of the rings of the precursor molecule (7-dehydrocholesterol) is broken down by ultraviolet B-light (UV-B, sun light). In part this explains why, when discovered a century ago, it was classified as vitamin. Actually, as previously noted, this substance is a member of a group of steroid molecules (secosteroids) with a common A, B, C and D ring structure derived from the cyclo-pentano-perhydrophenanthrene ring structure, very similar to cholesterol [13].

After the UV-B activity, this compound undergoes a very complex metabolic process that is controlled by classic endocrine feed-back mechanisms and becomes a biologically active hormone. It must be first hydroxylated in the liver, at the carbon 25-position by 25-hydroxylase, to form 25(OH)D, known as a calcidiol or calcifediol. Several cytochrome P450 (CYP) isoforms (including the mitochondrial CYP27A1 and the microsomal CYP2R1, CYP3A4 and CYP2J3) accomplish this hydroxylation step. CYP2R1 is thought to be the high-affinity 25-hydroxylase [14]. The 25(OH)D form is the most plentiful and stable pre-hormonal metabolite of vitamin D in human serum with a high affinity to bind serum vitamin D binding protein (VDBP) and other albumin superfamily proteins in the blood. As such, the 25(OH)D level in the serum is the best indicator of vitamin D entering the host, either by cutaneous synthesis or by ingestion in the diet. Nevertheless, this 25(OH)D form is still not a hormone, rather a pre-hormonal form of the natural hormone, and does not exert any biological activity in the body [15].

Calcidiol (25(OH)D) is then transported through the bloodstream, bound to vitamin D binding protein (VDBP), to the proximal tubule of the kidney, where it is hydroxylated at the 1α-position to form the final biologically active form of D hormone named calcitriol (1α,25(OH)2D), by the enzyme 25-hydroxyvitamin D-1α-hydroxylase (CYP27B1) [16]. Activity of this enzyme is increased by parathyroid hormone (PTH) secreted by the parathyroid gland, which is the pivotal activator of CYP27B1 in proximal tubule cells and decreases with aging [17]. Thereafter, the synthesized calcitriol becomes the real D hormone with full biological activity similar to other steroid hormones. The D hormone increases intestinal calcium absorption and mobilizes calcium from the skeleton while calcium level in blood regulates PTH secretion and CYP27B1 activity. These activities are clear examples of endocrine regulation due to D hormone production.

### D hormone biological activity

For many years it was believed that regulation of calcium homeostasis within the body with a positive influence on bone turnover were the only crucial roles of this hormone. These roles were why it was considered to be a vitamin critical for bone health. This tenet remains correct, but it is now understood that all monocyte-macrophage derived cells, including those present in many tissues and various epithelia, are able to express 1α-hydroxylase and to synthesize calcitriol locally, if there is a availability of the 25(OH)D substrate [18]. Synthetized D hormone can act on cells and in the tissues in an autocrine or paracrine manner, and the synthesized D-hormone (calcitriol) serves as connection between extracellular stimuli and genomic response of the cells [19].

It is recognized that 1α,25(OH)2D has high affinity to bind vitamin D receptor (VDR) due to the presence of an OH group at the 1α position. The VDR gene shows its highest expression in tissues with high metabolic activity, such as kidneys, bone and gut, but has low to moderate expression in nearly all other human tissues. VDR, when bound to hormone, heterodimerizes with the retinoic acid-X-receptor (RXR) and this complex binds to the vitamin D responsive element (VDRE) acting as a transcriptional factor to enhance or repress gene transcription [20].

It has been estimated that at least 200 tissues and as many as 2000 genes are directly or indirectly controlled by this transcriptional complex [21]. Only high doses of D hormone can induce genetic effects including immunomodulatory actions [20] while physiological actions have to be mediated via the genetic and epigenetic regulatory actions of the VDR transcriptional complex [22]. VDR protein has been detected both in the cytosol (associated with sarcoplasmic reticulum Ca2 + −ATPase) and in plasma membranes. This ubiquitous presence of the VDR protein may explain some of the rapid non-genomic actions of 1α,25(OH)2D such as calcium up-take that are related to calcium homeostasis and bone mineralization [23].

The signaling pathways of all steroid hormones (glucocorticoid, sex hormones) occur through cellular and nuclear hormone receptors [25]. All of these hormones influence bone formation and immune regulation. Steroid nuclear receptors, when bound to their agonist hormone, under control of co-regulators, catalyze or mediate chromatin remodeling, epigenetic modification, receptor recycling, and ultimately gene expression [26]. Gene regulation appears to be modulated by dual modifications of histone acetylation and DNA methylation. The 1α,25(OH)2D hormone has been shown to be a potent genetic and epigenetic regulator. This could be explanation for the possible pathogenetic role of low vitamin D status in immune-mediated diseases [24, 25].

Using the same signaling pattern, 1,25(OH)2D, locally produced in the tissues, exerts its effects on several immune cells, including macrophages, dendritic cells (DCs), T and B cells.

Macrophages and DCs constitutively express vitamin D receptor (VDR), whereas VDR expression in T cells is up-regulated after activation [27]. In macrophages and monocytes, 1,25(OH)2D positively influences its own effects by increasing the expression of VDR and the cytochrome P450 protein CYP27B1 (autocrine regulation). Toll-like-receptor (TLR)-mediated signals can also increase the expression of VDR.

The 1,25(OH)2D hormone also induces monocyte proliferation and production of interleukin-1 (IL-1) and cathelicidin (an antimicrobial peptide) by macrophages, thereby contributing to innate immune response [28, 29]. The 1,25(OH)2D hormone decreases DC maturation, inhibiting up- regulation of the expression of MHC class II, CD40, CD80 and CD86. In addition, it decreases IL-12 production of DCs and induce production of IL-10.

In T cells, 1,25(OH)2D decreases the production of IL-2, IL-17 and interferon-γ(IFNγ) and attenuates the cytotoxic activity and proliferation of CD4+ and CD8+ T cells [30]. The 1,25(OH)2D hormone might also promote the development of forkhead box protein 3 (FOXP3) + regulatory T (TReg) cells and IL-10-producing T regulatory type 1 (TR1) cells [31,32]. Finally, 1,25(OH)2D blocks B cell proliferation, plasma-cell differentiation and immunoglobulin production [33].

It is clear that the D hormone exerts its effects on many crucially important immunoregulatory proteins and cells [34]. Some of them are recognized as possible causative immune factors for the development of PRDs. Due to the D hormone’s proven capability to induce tolerogenic immune response, improve impaired T and B cell function, and enhance innate immunity response, a deficiency or insufficiency of D hormone may well have causative or risk factor effects [35] in pediatric rheumatic diseases.

### D hormone and pediatric rheumatic diseases

Numerous studies suggest that the 1,25(OH)2D hormone appears to play important roles in the pathogenesis of several autoimmune diseases, such as diabetes type I, multiple sclerosis and rheumatoid arthritis. Vitamin D deficiency has been found as a potential risk factor for increased cardiovascular risk, abnormal HDL and LDL cholesterol levels, hypertension, hyperglycemia, diabetes [36–41]. As this hormone affects bone and the immune system in many profound ways, pediatric rheumatologists cannot ignore its potential roles in PRDs.

- *Bone health in children*

Children and adolescents need to achieve peak bone mass by age 18 years or enter adulthood with suboptimal bone mass and risk of osteoporosis as adults [42]. The inflammatory nature of PRDs may reduce the ability to achieve peak bone mass [43] due to the inflammation, pain, decreased activity, and other factors. The necessity to use glucocorticoids for the treatment of pediatric rheumatic diseases is an additional risk factor for bone mass loss during childhood and adolescence [44]. It has been shown that patients with JIA and jSLE and other chronic inflammatory diseases have an increased fracture risk [45]. Glucocorticoids may have a regulatory effect on vitamin D metabolism which, in the presence of low vitamin D levels, may additionally negatively affect bone turnover [46].

The US Centers for Disease Control and Prevention (CDC) reported in 2006 that healthy children in the US are vitamin D deficient in approximately 9–11 % at age 1–8 years, 19–22 % at age 9–13 years and 22 % at age 14–18 years [47]. The possible significance of vitamin D deficiency in JIA was noted more than 20 years ago [48, 49]. Recently Pelajo et al. [50] reported that 20 % of all children attending clinic were vitamin D deficient while children with autoimmune disorders had a 2–3 fold greater probability of being vitamin D deficient compared to children with non-autoimmune conditions. Soybilgic et al. [51] assessed practices of North American pediatric rheumatologists regarding monitoring, prevention, and treatment of low bone mineral density (BMD) in children on long-term glucocorticoid treatment and found that 79 % "rarely" or "never" obtained a baseline BMD measurement prior to initiation of glucocorticoid therapy. Yet despite the lack of BMD assessment, 93 % "frequently" or "always" prescribed calcium for patients on long-term corticosteroid therapy, 81 % "frequently" or "always" prescribed vitamin D, and 40 % of the survey responders prescribed combined calcium/vitamin tablets.

It is very important to note that there are no data about vitamin D formulation and doses used routinely by pediatric rheumatologists. This may be partly due to the fact that there are no clear recommendations concerning this issue for pediatric rheumatic diseases. In adults, the Endocrine Society has recommended two to three time higher doses of vitamin D supplementation for adults treated with glucocorticoids than for the healthy population [52].

b) *Juvenile Idiopathic Arthritis (JIA)*

Interest and knowledge about vitamin D in JIA has increased in last decade and deficiency or insufficiency has been considered as important environmental determinant of the disease [53]. As mentioned, several studies have shown that vitamin D deficiency is associated with rheumatoid arthritis (RA) and type 1 diabetes (T1D) and that supplementation may be inversely associated with the disease onset [9, 54, 55]. Unfortunately these types of studies are missing for JIA and other pediatric rheumatic diseases. Recent meta-analysis performed by Nisar et al. [10] included 19 papers reporting values of 25(OH)D and 1,25(OH)D (14 and 11, respectively) in JIA, using similar assays, but could not find evidence to link vitamin D deficiency with JIA due to the lack of agreed definition of vitamin D deficiency in pediatric population. The main biases of this meta-analysis were that it did not use cut-off definitions for insufficiency and deficiency and the heterogeneity of study designs included in the meta-analysis. Nevertheless it is an important and useful overview and provides further evidence of the relatively high prevalence of vitamin D deficiency in JIA, especially in the systemic and polyarticular JIA catagories in which children are often treated with steroids. Mean levels of 25(OH)D within groups studied were 24.56 ng/ml (range, 11.5–56.4 ng/ml). These results are in agreement with a recent study of Pelajo et al. [56] who have found vitamin D deficiency (≤19 ng/ml) in 13 % and insufficiency (20–29 ng/ml) in 42 % of JIA patients, including those who were supplemented. The same authors could not find any association of 25(OH)D levels with disease activity (measured with JADAS-27), except in a subgroup of new-onset JIA who had non-significant negative correlation. Additional data about 25(OH)D levels and the influence on disease activity have been provided by a recent publication of Stagi S et al. [57]. These authors have confirmed significantly reduced 25(OH)D levels in JIA patients. In their study patients with active disease and/or frequent relapses have had significantly reduced 25(OH)D levels compared to patients with no active disease or infrequent flares. Two other studies [58, 59] have found that the percent true calcium absorption is below normal in children with JIA, while vitamin D supplementation (2000 IU) did appear to increase serum 25(OH)D and normalize serum calcium levels even without calcium supplementation.

c) *Juvenile Systemic Lupus Erythematosus (jSLE) and Dermatomyositis (JDM)*

High prevalence of vitamin D deficiency was shown in adult SLE but recent studies [60, 61] demonstrated that, despite vitamin D supplementation, deficiency is present in juvenile SLE.

Furthermore, vitamin D deficiency strongly correlated with SLEDAI, C4 level and BMD (low spine and whole body) [60]. Stagi et al. [61] has shown that jSLE patients exhibit lower 25(OH)D levels than controls with the lower values observed in patients with active vs. inactive disease.

Urinary losses of 25(OH)D and vitamin D binding protein (DBP) could be a reason for the low vitamin D status in pediatric lupus patients [62]. Robinson et al. reported vitamin D deficiency in jSLE patients, an inverse relationship between 25(OH)D levels and proteinuria, and an association with proliferative glomerulonephritis in patients with jSLE [63]. The same group published very interesting data as results of the APPLE study (Atherosclerosis Prevention in Pediatric Lupus Erythematosus) [64]. Briefly, the study confirmed that vitamin D deficiency is common in jSLE and independently associated with elevated hsCRP [11].

Additionally, jSLE patients with serum 25(OH)D ≥ 20 ng/mL had less mean-max CIMT (carotid intima medial thickness) progression following 3 years of atorvastatin treatment that could suggest that vitamin D deficiency may contribute to heightened inflammation and cardiovascular risk [65].

Data about vitamin D levels in JDM is very limited. Two studies, both including a small number of patients, observed that 25(OH)D level to be lower among children with high disease activity compared to low disease activity JDM patients [48, 63]. One recent study confirmed a significant association of low 25(OH)D serum levels with idiopathic inflammatory myopathies, including JDM [66].

It is very interesting to point out that association of vitamin D deficiency with pediatric rheumatic diseases may not be the only biologically important D hormone pathway. Polymorphism of genes regulating D hormone synthesis may be connected with the presence or severity of many rheumatic diseases [67]. Polymorphisms in vitamin D pathway related genes has been found to be associated with increased likelihood of being vitamin D deficient [68]. Ellis et al. [69] recently published data about impressive evidence of gene epistasis among all genes (GC, VDR, CYP24A1, CYP2R1, and DHCR7) regulating D hormone synthesis as well as the PTPN2 gene which is a vitamin D responsive gene determining susceptibility to JIA and type 1 diabetes. Several studies indicated a possible association of VDR receptor gene polymorphism with RA and JIA [70, 71]. In our study, we have found that presence of f variant of FokI VDR polymorphism was associated with a worse outcome and a longer need for biologic treatment in JIA patients [70].

### Optimal D hormone levels

The best method to determine a person’s vitamin D status is to measure the circulating level of 25(OH)D. Serum levels of 1α,25(OH)2D are often normal or even elevated in both children and adults who are vitamin D deficient due to its very short half-life and tight physiological control by PTH which can increase renal production of calcitriol (by stimulating 1α-hydroxylase activity). The vitamin D hormonal form is synthetized and accumulated to a large degree in the tissues but there it cannot be measured [15].

For a long time there has been no consensus on the optimal concentrations of serum 25(OH)D. Most authors have used the cut-off values of 10–15 ng/mL to define vitamin D deficiency. In 2010 the Institute of Medicine (IOM) concluded that vitamin D deficiency should be defined as a 25(OH)D level of <20 ng/mL for children and adults [72]. Based on a study in postmenopausal women in whom a rise of 25(OH)D level from ~20 ng/mL to ~32 ng/mL increased their efficiency of intestinal calcium absorption by 65 %, the Endocrine Clinical Practice Guidelines Committee of the Endocrine Society proposed a new definition of vitamin D insufficiency and sufficiency [52]. Vitamin D deficiency is now defined as 25(OH)D <20 ng/mL, vitamin D insufficiency as 21–29 ng/mL, and vitamin D sufficiency as >30 ng/mL for both children and adults. It is suggested that maintenance of a 25(OH)D level between 40 and 60 ng/mL is ideal and up to 100 ng/mL is safe [73]. These recommendations are summarized in Table 1. In adults it was shown that supplementation of 1000 IU of cholecalciferol per day increases 25(OH)D level by 7-10 ng/ml and it is believed that 100 IU can increase 25(OH)D level by as much as 2–3 ng/ml when serum 25(OH)D is below 15 ng/ml [74].

**Table 1:** Recommendations for patents at risk for D deficiency (JIA and other rheumatic inflammatory diseases)

Age	IOM recommendations for healthy children			IOM recommendations for healthy children at risk of vitamin D deficiency		Proposed recommendation for children with rheumatic diseases
	EAR IU (μg)/day	RDA IU (μg)/day	UL IU (μg)/day	Daily requirement IU/day	UL, IU/day	IU/day
Infants						
0–6 months	400 (10)		1000 (25)	400–1000	2000	1000
6–12 months	400 (10)		1500 (38)	400–1000	2000	1500–2000
Children						
1–3 years	400 (10)	600 (15)	2500 (63)	600–1000	4000	2000
4–8 years	400 (10)	600 (15)	3000 (75)	600–1000	4000	2000
Boys						
9–13 years	400 (10)	600 (15)	4000 (100)	600–1000	4000	2000
14–18 years	400 (10)	600 (15)	4000 (100)	600–1000	4000	2000
Girls						
9–13 years	400 (10)	600 (15)	4000 (100)	400–2000	4000	2000–3000
14–18 years	400 (10)	600 (15)	4000 (100)	400–2000	4000	2000–3000

Adapted from references 52 and 72; EAR-estimated average requirement; RDA-recommended daily allowance; UL-tolerable upper intake level

**Fig. 1:**
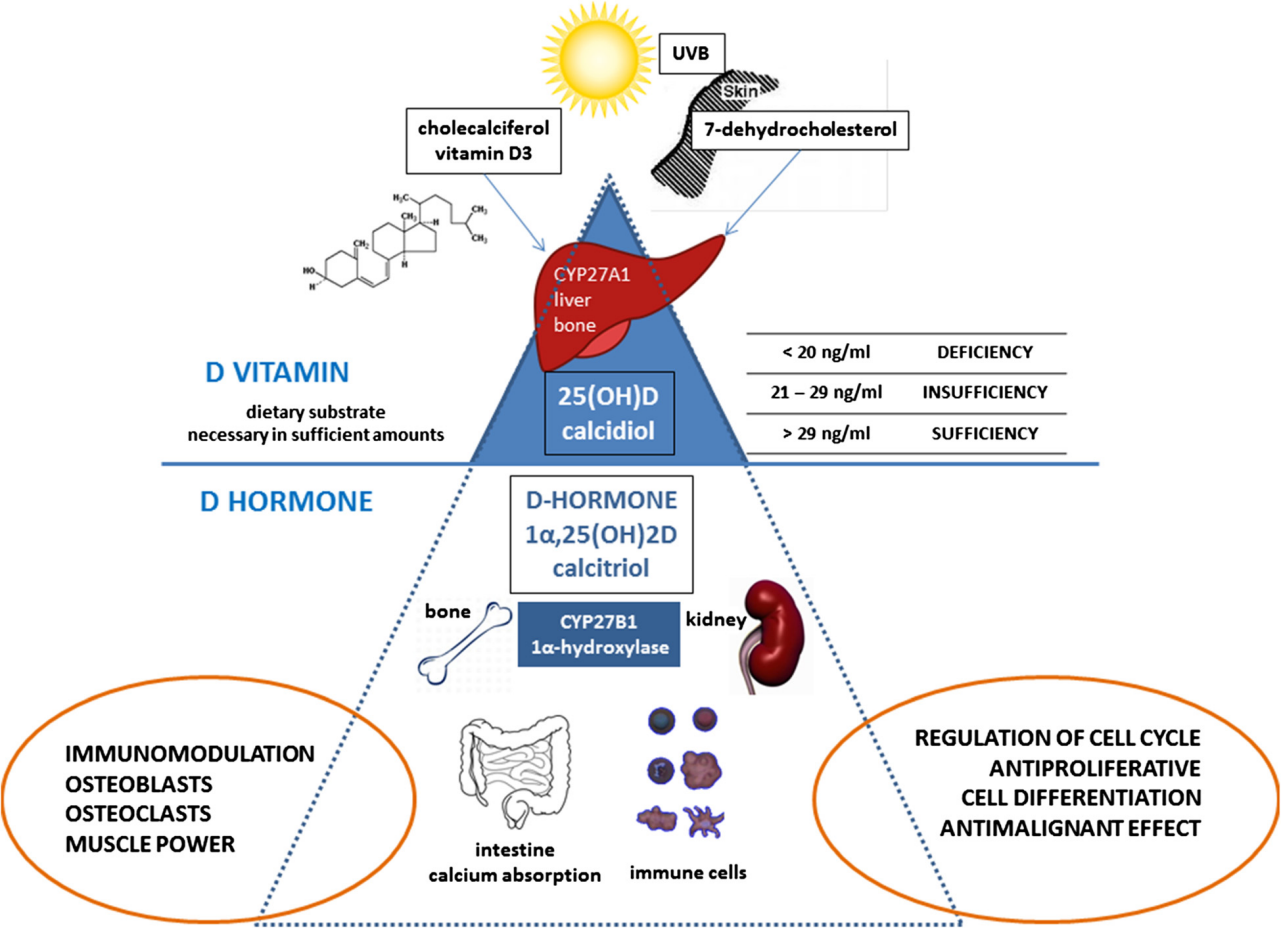
Vitamin D level estimation

As IOM recommendations are designed for healthy individuals of all ages, their direct application in children with JIA or other rheumatic diseases could be challenging especially due to long term steroid usage and high disease activity. These adverse effects seen in JIA and other rheumatic diseases argue for higher supplementation doses in these children [52]. It is of great importance for physicians to understand that measured 25(OH)D level is a reflection of food and/or supplement intake, but at the same time it can be a result of increased utilization and need for this pre-hormonal substrate in the local tissues (especially immune cells and bone) in the state of chronic inflammation (Fig. 1). That is why, for children with rheumatic diseases, we recommend pediatric rheumatologists to use at least doubled daily allowance doses (RDA) of cholecalciferol as vitamin D supplementation, especially for patients treated with glucocorticoids (Table 1).

As discussed earlier, supplementation with vitamin D (cholecalciferol) is unfortunately not common in everyday pediatric rheumatology practice [51]. Additionally there is huge variety of formulations (ergocalciferol or cholecalciferol, with or without calcium) used in multiple different dosing regimens. Cholecalciferol (D3) should be the preferred form for supplementation as it was shown that it can yield greater increases in 25(OH)D compared to an equivalent dose of ergocalferol (D2). Additionally, 25(OH)D is the required substrate for conversion to1,25(OH)2D at the cellular level and D2 (ergocalciferol) does not have the long-term potency as D3 [75]. Supplementation with calcitriol (1,25(OH)2D – final hormonal form) or other vitamin D analogues is generally recommended only for children with chronic kidney disease, patients taking anticonvulsants or suffering from malabsorption syndromes. The patients are usually unable to metabolize cholecalciferol to the hormonal form [76].

Among pediatric rheumatologists, there may be fear that using higher vitamin D doses could carry a risk of hypercalcemia and toxicity. We doubt this concern is justified since infants who received the huge doses of 200,000–600,000 IU of vitamin D2 or vitamin D3 orally for vitamin D deficiency have had no reports of toxicity. Rather neonates treated with 2000 IU of vitamin D3 during the first year of life appeared to have a reduced risk of developing an autoimmune disease, ie type 1 diabetes, and did not experience toxicity [54]. Also, the risk of hypercalcemia can be lowered if additional calcium intake is avoided since it has been shown that calcium has limited potential to effect bone acquisition [59].

D hormone as a natural compound is necessary to be present in high concentrations in cells and tissues to achieve genetic and epigenetic effects. This is why pharmaceutical companies focused on the development of more than 3000 D hormone analogs (agonists) [8]. There is some evidence that vitamin D analogs can even overcome steroid resistance [77] and improve disease outcome [78] but randomized controlled trials and other studies are needed.

## Conclusion

Vitamin D deficiency has clearly been recognized as pandemic and connected with numerous non-infectious diseases with increased incidence in the modern age (cardiovascular, malignant, autoimmune, cognitive etc.) and even increased risk of mortality [74, 79–81]. From epidemiological studies it is clear that vitamin D deficiency is associated with numerous diseases but it is not totally clear if it is a cause or a effect. Due to disease and medication effects, children with rheumatic diseases have additional needs for vitamin D supplementation. It is reasonable to assume that a child with a rheumatic disease, especially if treated with steroids, needs at least double the daily recommended dose of vitamin D for age (approximately 2000 UI/day) [82]. This high dose is needed as immunomodulatory and epigenetic benefits from 1,25(OH)D (hormonal form of vitamin D) can be achieved only when high levels are present in the tissues [24]. Vitamin D supplementation is an appealing adjunct treatment option in JIA and other inflammatory rheumatic diseases due to its pleiotropic effects, which may both minimize bone fragility and attenuate the immune hyperactivation. It is clear that it is now of great importance that pediatric rheumatologists use vitamin D supplementation for children with rheumatic diseases as well as systematically collect data about vitamin D and disease severity and outcome that will inform Vitamin D guidelines in the future.
